# Factors affecting self-perceived mental health in the general older population during the COVID-19 pandemic: a cross-sectional study

**DOI:** 10.1186/s12889-024-18199-1

**Published:** 2024-03-01

**Authors:** Jenny Koppner, Ann Lindelöf, Fredrik Iredahl, Maxine Tevell, Staffan Nilsson, Annika Thorsell, Åshild Faresjö, Hanna Israelsson Larsen

**Affiliations:** 1 Department of Health, Medicine and Caring Sciences, Division of General practice, Linköping University, Linköping, Sweden; 2 Department of Health, Medicine and Caring Sciences, Division of Society and Health/Public Health, Linköping University, Linköping, Sweden; 3 Department of Biomedical and Clinical Sciences/Center for Social and Affective Neuroscience, Medicine, Linköping University, Linköping, Sweden

**Keywords:** Anxiety, COVID − 19, Depression, Family situation, Mental health, Older adults, Physical activity, Primary health care, Social life, Stress

## Abstract

**Background:**

Mental health problems among older people are large public health concerns but often go unrecognized and undertreated. During COVID − 19 several restrictions regarding social contacts were launched, primarily for the old. The objective of this study is to investigate which factors that had the main negative affect on mental health in the older population during the pandemic.

**Method:**

A cross-sectional cohort study set in Swedish primary care during the pandemic years 2021–2022. The population constitutes of 70–80-years-old, *N* = 260. Instruments used are Geriatric depression scale 20 (GDS20); Hospital anxiety and depression scale (HADS), and Perceived stress scale 10 (PSS10). Sociodemography and risk factors are explored. Outcome measures are factors independently associated with decreased mental health. Analyses were performed for the group as a whole and with logistic regression models comparing individuals who stated they were mentally affected by the pandemic to individuals who stated they were not.

**Results:**

Participants who stated they were mentally affected by the COVID − 19 pandemic reported significantly higher levels of anxiety (*p* < 0.001), depression (*p* < 0.001), and stress (*p* = 0.026) compared to those who stated they were not mentally affected. Explanatory regression models of up to 50% showed that following factors were prominent among individuals who reported a decline in their mental health due to the COVID − 19 pandemic (*n* = 24); impaired social life (OR 20.29, *p* < 0.001, CI 4.53–90.81), change in physical activity (OR 5.28, *p* = 0.01, CI 1.49–18.72), perceived family situation (OR 31.90, p = 0,007, CI 2,53–402.42), mild/moderate and high anxiety (OR 4.94, *p* = 0.034, CI 1.13–21.60, OR 7.96, *p* = 0.035, CI 1.16–54.53 respectively), and female gender (OR 6.52, *p* = 0.029, CI 1.22–34.92).

**Conclusion:**

Anxiety, family situation, social life and change in physical activity were the main factors influencing the 70–80-years-old’s self-perceived mental health during the COVID − 19 pandemic. Long-term effects of social restrictions on mental health in the older population need to be further investigated.

**Supplementary Information:**

The online version contains supplementary material available at 10.1186/s12889-024-18199-1.

## Introduction

The older population in the world is increasing; average life expectancy has increased globally for people aged 60 years and over due to progress in medical technologies, advancements in public health, and improved sanitation [1]. It is well known that this age group has a large burden of somatic disease, however, they also suffer mental health issues; depression is so common among older people that it can be seen as a public health problem [2]. Other common occurrences are scattered families, loss of close ones, less income, and poorer ability to move, which are risk factors for social isolation and loneliness [1]. Social relationships have a significant impact on overall longevity and physical well-being [3], whereas social isolation and feelings of loneliness are factors associated with higher morbidity and mortality as they affect psychobiological processes, leading to neuroendocrine dysregulation, inflammatory responses, chronic allostatic load, and disturbances in autonomic function [3]. Anxiety and depressive disorders also alter psychobiological processes, above all the stress systems, leading to an increased risk for the onset of chronic disease including physical and cognitive impairment [4].

During the winter of 2019/2020, the virus SARS-CoV-2 was discovered, commonly called COVID − 19. Because of the alarming levels of spread and severity of the virus, the World Health Organization characterized the situation as a pandemic on March 11, 2020 [5]. The COVID − 19 virus hit the older population the hardest. Studies have shown that high age was a major independent risk factor for severe disease and death [6]. The higher the age the higher the risk; aged 75–79 years, followed by aged 70–74 years constituted the majority of patients in intensive care, and had the highest death rates [7]. Because of this, the Public Health Agency of Sweden introduced several restrictions and recommendations in Sweden for people aged 70 years and older. They were recommended to limit their close contact with others, i.e. avoiding public gatherings, public transportation, stores, and public facilities, even contact with family and friends. The government also established a national ban on visits to nursing homes [8].

Previous research regarding the effects of the COVID − 19 pandemic on mental health in the older population has mainly focused on the early stages of the pandemic. A review from 2020 [9] discusses possible physiological mechanisms behind effects of covid 19 infection on mental health in older people. An increased vulnerability in this population is advocated. Another review [10] shows that this population is more likely to suffer mental health issues associated with COVID − 19, and that the older population reported e.g., stress, depression, anxiety, and loneliness during the pandemic. Comparable results were found in a Swedish study [11] among seniors in an urban setting. However, in a Swedish longitudinal study from 2021, where very early effects of the pandemic on 65–71-year-olds were investigated, results show an equal well-being in the population, or even higher, as before the pandemic [12].

In summary, there is a growing senior population in the world with many risk factors for decreased physical and mental health. Older people were the most vulnerable during the COVID − 19 pandemic and they also were one of the population groups most affected by severe restrictions regarding social contacts. Now that we are in the aftermath of the pandemic, it is important to evaluate the consequences of these restrictions to learn for the future [13]. The aim of this study was to investigate which factors affected mental health among 70–80-year-olds during the COVID − 19 pandemic.

## Materials and methods

### Study design

**Fig. 1:**
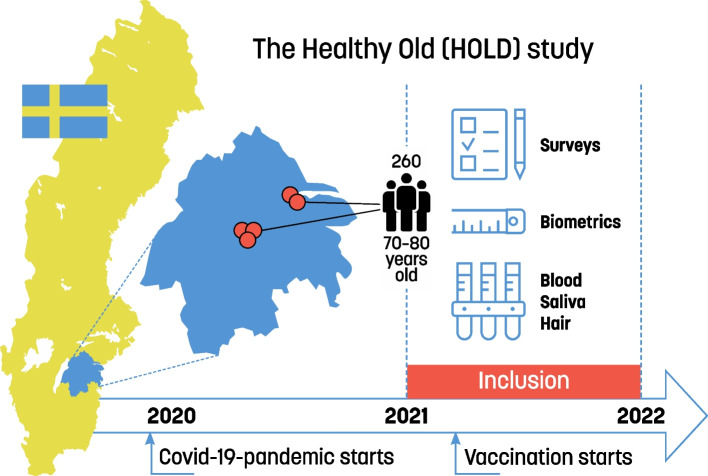
Overview of the HOLD study protocol. In the HOLD-study 260 inhabitants in the Southeast of Sweden, between the age of 70–80 years, were randomly asked to participate. At the inclusion subjects were studied in regards of biometric data, surveys, blood samples, hair sample, and saliva

This is a cross sectional study, in which data was collected within the HOLD- (Healthy OLD people) study situated in the southeast of Sweden, Region Östergötland, as visualized in Fig. 1. The HOLD-study aims to identify important factors to focus on for promoting healthy aging, defined according to the WHO [14], and in doing so investigate long-term stress, psychosocial factors, and health factors in the increasing older population in Sweden.

### Participant recruitment

Five Primary Health Care centers (PHC) were included, situated in both rural and urban locations. Inclusion criteria were age 70–80 years old and being listed at one of the PHCs, thus, all listed 70–80 years old at each PHC were eligible for recruitment (*N* = 7796). All individuals were placed in random order via Excel on a list and the first 100 on the list were initially offered to participate in the study. The goal was to recruit at least 60 participants at each PHC with an exception for one PHC included late in the process, thus having a much shorter time for recruitment. If that aim was not reached from the first 100 on the list recruitment continued from the 101st and onwards until the goal was met. All in all, two PHCs recruited 64 and 60 participants respectively, whereas two PHCs were excepted from the goal of 60 individuals, due to the time limit of the HOLD-study, and a smaller sample was accepted (55 and 52 respectively). The fifth PHC, included last, recruited 29 participants. Thus, in total 260 participants were recruited, which succeeded the goal of 240. Information about the study, including a consent form, was mailed to the participants, for them to read and reflect upon whether they would like to participate. Shortly thereafter they received a phone call from a research nurse. Phone numbers were searched for on public digital services. Exclusion criteria in the HOLD-study were: living in a nursing home, inability to find the phone number on public digital services, the participant not answering the phone after three call attempts, inadequate cognitive skills, inadequate written and/or oral Swedish language skills, no hair or hair shorter than 1 cm on the scalp (for biochemical analyses in other HOLD sub studies), participants without ability to visit the PHC. If all requirements were met, and the person wanted to participate in the study, an appointment was booked at the PHC for signing the written consent form and collection of data. If the presumptive participant did not come to the appointment without giving notice, he/she was excluded. There was no difference between included and excluded groups regarding sex (*p* = 0.50), and age (*p* = 0.36). There were no dropouts once the written consent form was signed.

### Data collection

The study was conducted from October 2021 to December 2022. All data was collected by the research nurse during the visit to the PHC. Biometric values were collected (weight, height, waist measurement, blood pressure and pulse), as well as samples of blood, hair, and saliva, these were then used for other sub studies of the HOLD-study. All participants answered a questionnaire that consisted of 29 segments. Questions included sex, age, and self-perceived: financial status, social life, loneliness, physical activity, and health, as well as effects of the COVID − 19 pandemic, including some perceived differences before and during the pandemic. The participants were also asked to choose up to two factors from a given list that they felt had affected them the most by the Covid − 19 pandemic. The question about self-perceived health in the survey was based on the Short Form Health Survey (SF-36) which is a common instrument for measuring quality of life and health [15]. Following three screening scales, all validated in Swedish, were used to assess the participants’ mental health. Perceived Stress Scale-10 (PSS-10); 10 questions that measure a person’s level of stress during the last month, with a total score of 0–40, where a high score indicates a high level of stress [16]. Hospital Anxiety and Depression scale (HADS); a diagnostic tool that evaluates both anxiety and depression consisting of seven questions about depression and seven questions about anxiety, with a total score of 0–21 respectively then divided into three categories: score 0–7 no depression/anxiety, score 8–10 possible depression/anxiety, score > 11 indicates depression/anxiety [17], Geriatric Depression Scale-20 (GDS-20); a screening instrument intended to identify depression in elderly people capturing how the patient is feeling now and during the last 2 weeks with 20 yes-or-no questions, with a total score of 0–20 then divided into two categories: 0–5 = depression is not likely, 6–20 = depression can be suspected [18]. For all three screening scales both total score and division into categories were used in this study.

### Statistical analysis

**Fig. 2:**
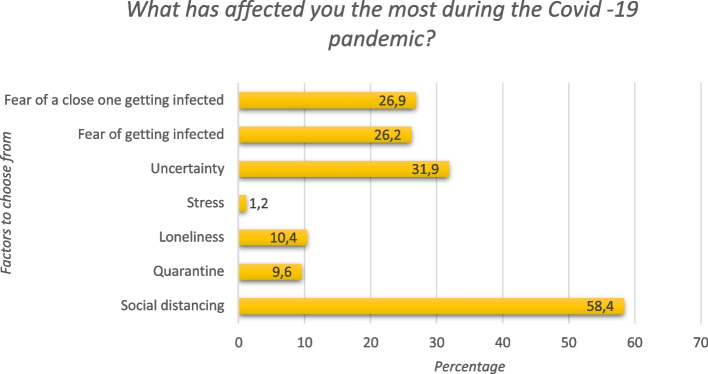
Factors affecting the participants the most during the COVID − 19 pandemic. Distribution of factors chosen by participants as those that affected them the most during the COVID − 19 pandemic. Each participant was asked to choose up to two, out of seven, options

All data was stored in a common database and statistically analyzed using the SPSS version 28.0 software (SPSS Inc., Chicago, IL, USA). For descriptive statistics, the frequencies were conducted for all variables. Chi^2^-tests were also conducted for all variables. For the question “What has affected you the most?”, frequencies were displayed in Fig. 2. The factors that the participants reported had affected them the most during the pandemic were calculated. The self-rating scales were transformed into numeric values and categorized. Nonparametric tests Mann Whitney and Kruskal Wallis were used to obtain differences between affected and not affected. Correlations were investigated between “Has your mental health been affected by the COVID -19 pandemic?” and the variables: sex, age, education level, civil status, financial status, family situation, changes in social life, changes in feelings of loneliness, changes in physical activity, GDS-20 divided into groups, HADS depression (HADS-D) divided into groups, HADS anxiety (HADS-A) divided into groups, and PSS-10 divided into groups using Spearman’s correlation. Separate analyses for men and women were also performed. In the final binary logistic regression model only yes- and no-answers were included for the dependent variable “Has your mental health been affected by the Covid –19 pandemic”. The level of statistical significance was set at *p* = 0.05.

## Results

In total *N* = 260 participants were included in the study. Demographic data is presented in Table 1. A majority of the participants were women, and the most common civil status was married or cohabited. Education levels were quite equally distributed between secondary school, university/college, and primary school. More than 90% of the participants felt that their financial status was good, and almost as many participants claimed the same regarding their family situation. Most of the participants reported a good social life, and that they rarely felt lonely, often participated in social activities, and perceived their health as good.

**Table 1:** Participants’ self-reported characteristics in total and divided into gender

Variables	Total *N* = 260% (n)	Men *n* = 114% (n)	Women *n* = 146% (n)
**Civil status**			
Single	28% (72)	20% (19)	34% (49)
Married/cohabited	72% (186)	80% (90)	66% (96)
**Education**			
Primary school	29% (76)	30% (20)	30% (42)
Secondary school	35% (88)	34% (38)	35% (50)
University/college	35% (90)	36% (41)	35% (49)
**Financial status**			
Good	94% (142)	97% (110)	92% (32)
Bad	6% (15)	3% (4)	8% (11)
**Family situation**			
Good	92% (236)	94% (106)	91% (30)
Bad	2% (6)	2% (2)	3% (4)
Neither good nor bad	6% (14)	4% (5)	6% (9)
**Social life**			
Good	86% (222)	83% (95)	88% (127)
Bad	2% (5)	2% (2)	2% (3)
Neither good nor bad	12% (21)	15% (17)	10% (15)
**Feeling of loneliness**			
Rarely	96% (249)	98% (112)	95% (137)
Often	4% (10)	2% (2)	5% (8)
**Participating in social activities**			
Often	74% (191)	73% (83)	75% (108)
Rarely	26% (66)	27% (22)	25% (23)
**Physical activity**			
Regularly	85% (218)	80% (90)	89% (128)
Not regularly	15% (39)	20% (19)	11% (16)
**Self-perceived health**			
Good	87% (225)	90% (103)	84% (122)
Bad	13% (20)	10% (11)	16% (19)

As shown in Table 2, only a few participants have had confirmed COVID − 19 disease themselves, but many have had a close relative who has been sick with COVID − 19. Almost every 10th person felt that their mental health was affected by the pandemic, and slightly more reported that they did not know. When asked to choose up to two factors, from a given list, that affected them the most during the COVID − 19 pandemic, social distancing was the most common, followed by a feeling of uncertainty, and fear of a close relative, or themselves, catching COVID − 19 infection (Fig. 2).

**Table 2:** Participant characteristics regarding the COVID − 19 pandemic

Variables	Total % (n)	Men % (n)	Women % (n)
**Have you had a confirmed COVID − 19 infection?**			
Yes	13% (24)	11% (12)	16% (19)
No	82% (211)	84% (95)	80% (116)
Do not know	5% (12)	5% (6)	4% (6)
**Has a close relative to you had confirmed Covid − 19 infection?**			
Yes	70% (180)	64% (72)	74% (108)
No	25% (65)	28% (21)	23% (25)
Do not know	5% (12)	8% (9)	3% (5)
**Has your mental health been affected negatively by the Covid − 19 pandemic?**			
Yes	9% (26)	4% (5)	13% (27)
No	79% (204)	88% (99)	72% (105)
Do not know	11% (22)	8% (9)	15% (28)
**Self-perceived change in feeling of loneliness during the COVID − 19 pandemic**			
Negative change	9% (26)	4% (5)	13% (27)
Positive change	4% (10)	4% (4)	4% (6)
No change	87% (225)	92% (105)	83% (120)
**Self-reported change of social life during the COVID − 19 pandemic**			
Negative change	14% (23)	11% (13)	16% (19)
Positive change	6% (15)	7% (8)	5% (7)
No change	80% (207)	82% (93)	79% (114)
**Self-reported change of physical activity during the COVID − 19 pandemic**			
No change	77% (199)	87% (99)	69% (100)
Change	23% (61)	13% (15)	31% (46)

Depression, anxiety, and stress were all common among the participants, distributed as follows: as many as 64% (*n* = 174) met the scores for likely depression in GDS-20, but in HADS-D only 2% (*n* = 5) reached risk of depression. However, in HADS-D 76% (*n* = 197) were categorized as dejection. Every 5th participant (*n* = 52) reported mild to moderate anxiety in HADS-A, and 5% (*n* = 13) high such. The distribution of self-reported stress was: low stress 0.4% (n = 1), moderate stress 71.8% (*n* = 186), and high stress 27.8% (*n* = 72) among the total study population.

As shown in Table 3, when comparing those who reported to be mentally affected by the COVID − 19 pandemic with those who did not feel mentally affected significant differences concerning GDS, HADS-D, HADS-A, and PSS scales could be seen among the affected compared to the non-affected. A third group, “do not know if affected” (*n* = 30), showed the same pattern as the affected group concerning these scales (*p* ≥ 0.48), see Table S1 in supplements.

**Table 3:** Mental health issues and self-reported effect on mental health by the COVID − 19 pandemic

Variables	*Yes, affected*		*No, not affected*		
	N	Mean rank	N	Mean rank	*P*-value
**GDS-20**	24	160	203	108	< 0.001
**HADS depression**	24	137	203	111	0.053
**HADS anxiety**	24	160	203	108	< 0.001
**PSS 10**	24	142	203	110	0.026

Comparison between self-reported negative effect on mental health by COVID −19 pandemic and rating scales detecting mental health issues in the study population, (divided into: yes, affected; no, not affected) and Geriatric Depression Scale 20 items (GDS-20), Hospital Anxiety and Depression scale (divided into HADS depression and HADS anxiety), and Perceived Stress Scale 10-items (PSS-10). Total scores were used for all screening scales. Mann Whitney-U test was used for analyses.

There was a correlation between participants perceiving their mental health having been negatively affected by the COVID − 19 pandemic (yes- or no-answers only) and following variables: sex where women were more affected than men (r = 0.171, *p* = 0.01), negative change in social life (r = 0.320, *p* < 0.001), worse family situation (r = 0.380, *p* < 0.001), a change in loneliness (r = 0.20, *p* = 0.002), change in physical activity (r = 0.353, p < 0.001), and higher scores on GDS-20 (r = 0.169, *p* = 0.01), HADS-A (r = 0.299, *p* < 0.001), and PSS-10 (r = 0.140, *p* = 0.35), see Table S2 in supplements. Similar results were shown when those who answered that they did not know if their mental health had been affected by the Covid − 19 pandemic was added to the correlation analysis. When comparing men and women, female gender was significantly correlated with single civil status (r = 0.168, *p* = 0.007), more feelings of loneliness r = 0.133, *p* = 0.03), changes in physical activity (r = 0.215, *p* < 0.001), higher scores on GDS-20 (r = 0.214, p < 0.001), and HADS-A (r = 0.164, *p* = 0.008).

In Table 4 a comparison is shown between the participants that reported deteriorated mental health due to the Covid − 19 pandemic and those that did not report such effect. Increased risks were seen among those affected, especially those who reported a bad family situation, those who reported a negative change in their social life, those with anxiety, and for women. Also, those who reported a change in physical activity had significantly changed mental health. The final binary logistic regression model explains up to 50% of the risk for deteriorated mental health during the COVID − 19 pandemic when exposed to various psychosocial factors.

**Table 4:** Risks for being affected by psychosocial factors during the Covid − 19 pandemic, comparing participants who reported a negative effect on their mental health due the pandemic to those who did not

Independent variables	*P*-value	OR (95% CI)
**Gender**		
Men	*Reference*	*Reference*
Women	0.029	6.52 (1.22–34.92)
**Family situation**		
Good	*Reference*	*Reference*
Neither good nor bad	0.004	17.15 (2.50–117.47)
Bad	0.007	31.90 (2.53–402.42)
**Change in social life during the COVID − 19 pandemic**		
No change	*Reference*	*Reference*
Negative change	< 0.001	20.29 (4.53–90.81)
Positive change	0.34	3.33 (0.28–38.92)
**Change in feeling of loneliness during the COVID − 19 pandemic**		
No change	*Reference*	*Reference*
Negative change	0.33	0.37 (0.05–2.76)
Positive change	0.57	1.79 (0.24–13.20)
**Change in physical activity during the COVID − 19 pandemic**		
No change	*Reference*	*Reference*
Change	0.010	5.28 (1.49–18.72)
**HAD anxiety**		
None – very low	*Reference*	*Reference*
Mild-moderate	0.034	4.94 (1.13–21.60)
High	0.035	7.96 (1.16–54.53)
**GDS − 20**		
No depression	*Reference*	*Reference*
Depression likely	0.71	1.37 (0.26–7.36)

A binary logistic regression model with “Has your mental health been affected negatively by the Covid –19 pandemic?” as dependent variable. The model is statistically significant with p < 0,001, df 11, Cox & Snell R square 0,272, and Nagelkerke R square 0,560

## Discussion

In summary, the most important findings in this study were that participants who perceived that their mental health had been negatively affected by the COVID − 19 pandemic had more symptoms of anxiety, depression/dejection, and stress. When analyzing risk factors for experiencing a decline in mental health during the pandemic, of these, only anxiety fell out as a risk factor, as well as a negative change in social life, a change in physical activity, experiencing a bad family situation, and female gender. Anxiety is common in the older population [19, 20], and during the COVID − 19 pandemic an increase was likely to occur as this age group was especially vulnerable to the virus, more socially isolated, and the special situation of a pandemic is associated with uncertainty regarding both the present and the future. Studies performed during the COVID − 19 pandemic confirmed increased anxiety among the older adults [10, 11, 21]. Our result reflects what other research have found and suggests that anxiety is of major concern when evaluating and predicting mental health in the older population during pandemic-like conditions.

The participants in this study reported that the factors affecting them the most during the COVID − 19 pandemic were social distancing, uncertainty, and fear of a close relative, or themselves, catching COVID − 19. Comparable results are found in previous studies, but few asked their participants such a direct question, and, thus, their results are concluded more indirectly [10, 21, 22]. However, to our surprise, the majority of our study participants did not perceive that the COVID − 19 pandemic had affected their mental health. This contrasts with previous studies. In two reviews from 2022 [10, 21] it is concluded that older adults are prone to suffer mental health issues due to the Covid 19 pandemic, e.g.: loneliness, depression, anxiety, and stress. One major reason for this dissimilarity may be due to a large majority of our participants living with a partner, and, thus, were not completely socially isolated, which might also explain why they reported a low degree of loneliness and perceived their mental health as good. In line with this hypothesis, a previous Swedish study found an association between decreased mental wellbeing and social isolation among older adults [11]. Another factor to consider is that Sweden did not apply lock downs, as most other nations including neighboring Nordic countries [23], and therefor loneliness and social isolation might be less of an influence on our population.

In Sweden it is estimated that approximately 300,000 people are socially isolated, pre-pandemic, which is defined as a person living alone, meeting relatives, friends, or acquaintances twice a month or less. After retirement social isolation increases, and in the age group 75–84 years every 10th person is socially isolated [24]. It is known that the pandemic increased social isolation in this age group and several studies show that this affected their mental health [10, 11, 21]. In accordance with this our study found that those who reported a deteriorating social life during the pandemic also suffered from declining mental health. Interestingly though, as many as 80% reported no change in their social life, and 6% reported a positive change, few lived alone, and they were doing well in general. A possible explanation could be that these participants adapted well to the pandemic changes. Supporting this are the findings of Özdemir and Çelen [25] who saw that increased stress among old adults was associated to social isolation and chronic disease, and Sardella et al. [26] whose results show that living with someone might be a factor of resilience. Studies also show that this age group have good psychological coping and adaptability, and that activities such as social media use, communication with others e.g. neighbors, seeking social support, and keeping themselves busy during the pandemic were successful protective measures [27, 28]. We did not ask specifically about our participants’ social media use or communications with others during the pandemic, but in Sweden internet access and use is high, and in the ages 70 and older up to 75% use social media daily, thus, it is possible that this has acted as a favorable factor in our studied population [29].

Previous study results show that, in general, older women suffer from more anxiety, fear, worry, depression, and depressive symptoms than older men [2, 17]. In this study we found a similar pattern with women being more negatively affected mentally by the pandemic than men. These findings were expected and are consistent with other research during the pandemic, e.g. a Canadian study who found that women were twice as likely to report depressive symptoms than men [30], and a Chinese study reporting that women were more affected by anxiety and stress [31]. Uniformly, there are findings suggesting that older women might be more vulnerable in facing the COVID − 19 pandemic than older men [26].

According to WHO, 14% of the world’s population aged 60 and over suffer from at least one mental health disorder, depression and anxiety being the most common, and more than a quarter of all deaths by suicide occur in this age group [19]. When comparing GDS 20, HADS-D and HADS-A to self-reported negative effect on mental health by the Covid − 19 pandemic in this study, symptoms of anxiety and stress in HADS-A, and PSS 10 fall out as strong predictors for declined self-perceived mental health due to the pandemic. Depression also falls out as a strong predictor, however, only in GDS 20 and not in HADS-D, which was somewhat surprising. But, in HADS-D a large majority scored as “dejection”, in fact the exact same percentage (76%) as those who scored “likely depression” in GDS 20. This discrepancy between the two screening scales is in line with the findings of G. Campbell et al. [3], and is probably because GDS 20 is designed especially for the older population whereas HADS is constructed for the adult population in general.

In contrast to other studies [32, 33], depression and stress do not fall out as a risk factors in this study. A reason for this might be, as discussed earlier, that most of our population lived in a relationship and few felt lonely, which could have increased their psychological resilience. One could also argue that stress levels might be lower later in the pandemic as fewer became severely ill after vaccinations started, restrictions lessened, and both the present and the future were less uncertain. However, our participants did state that social distancing and uncertainty were two of the factors that affected them the most, but apparently only a minority were so affected that they reported decreased mental health.

A major strength in this study is the use of reliable and well-validated screening scales that together capture different aspects of mental illness. In addition, there is good agreement between the self-rating scales, which further strengthens our result. Two different scales for depression were used, which we consider to be a strength. For anxiety only HADS was used. This could be considered a limitation. However, HADS is well renowned and used frequently for investigating anxiety, also in older people. Furthermore, our results regarding anxiety are very rigid and show consistent statistical significance in the different analyses, thus, we consider our results reliable and unlikely to change direction using another screening tool. Physical activity is known as an important factor for maintaining good mental health, and the same has been shown for older people during the pandemic [34, 35]. The results of this study indicate that a change in physical activity is a possible strong risk factor for declining mental health (*p* = 0.01, OR 5.28), however, the direction of change is unknown due to how the question was formed. This, of course, is a limitation. Regarding that our analysis found a correlation between change in physical activity and a decline in social life (r = 0.156, *p* = 0.012) indicates that the change is negative (i.e. reduced physical activity), but we cannot know for sure. Another limitation is the possible impact of recall bias affecting the result, a well-known phenomenon to take into consideration when using self-reported data, but, in general self-reports are considered reliable and well established [36]. Our focus on the question “Has your mental health been affected negatively by the Covid –19 pandemic?” for much of our analyses is a possible limitation, as it means that much of our analyses depends on a single, self-estimated question. However, it is common in research to investigate the subjective measure of participants’ perception, and it is also a natural approach in the doctor-patient consultation, since the perspective of how people perceive e.g. their well-being is valuable information both on an individual level (to help the patient) and on a societal level (develop strategies to promote health in a population). In this study we also complement the question in focus with validated screening scales measuring mental health that showed a significant difference between those who perceive a decline in their mental health due to the pandemic and those who do not. Considering this, we believe the focus on a single question can be motivated in this study. Another limitation here is the size of the sub-population answering this focus question “Yes” (*n* = 24), which motivates some caution when interpreting these results. Those who answered “Do not know” (*n* = 30) were considered to be included into this sub-population as sub-analyses showed that their mental health was nearly identical to those who answered “Yes”. This would have more than doubled the sub population size, and, thus, strengthened the analyzes. However, they were excluded since it might be that these participants had equally poor mental health before the pandemic, and, thus, their screening scores would not reflect a decline during to the pandemic. A strength to consider, though, is that the recruitment base of the HOLD-study, all 70–80 years old listed at the included PHCs, is likely to reflect the age group well since it consists of the general primary care population. Also, those eligible were approached from a randomized list of this general older population, and, thus, the studied population is probably representative for the population. However, it is important to note a couple of limitations considering the recruitment process and study population. According to exclusion criteria, individuals: living in nursing homes, not able to visit their PHC, with cognitive dysfunction, and those with inadequate language skill were excluded. This, in combination with the well-known phenomenon that healthy people are more likely to participate in research than those in poor health [37], means that frail elderly and immigrants might have been underrepresented in this study. Though, noticeably, only seven were excluded due to cognitive impairment or living in a nursing home, and five due to lack of language skills. This could reflect a less diverge sociodemographic uptake of the PHCs, but the included ones covered geographic areas with several nursing homes, and a diversity in income levels, education levels, and immigrant density. Therefore, a more probable explanation is that the underrepresented groups were less likely to embrace the information sent out or answer the phone, and thus were excluded on those criteria. All in all, the underrepresentation of frail elderly is likely to make the studied population here healthier than the total general older population, however, it is still noteworthy that among those who report a decline in mental health due to the pandemic anxiety, depression, and stress were significantly overrepresented. If our study population had been less healthy, thus, perhaps reflecting the general population better, we would probably have found more mental health issues. Observing this connection within the smaller group implies a likelihood that it is true.

## Conclusion

Risk factors for decreased mental health during the COVID − 19 pandemic detected in this study were a negative change in social life, a change in physical activity, experiencing a bad family situation, anxiety, and female gender. Surprisingly, depression and stress were not found to be risk factors. However, anxiety along with depression and stress were more common among those who stated that their mental health had been negatively affected by the pandemic compared to those who did not. In the clinic, this knowledge further emphasizes the importance of asking how the patient is affected by a difficult life event (pandemic or other), not only ask how they feel. However, more longitudinal studies regarding mental health issues in the older population are needed, in general and to better understand the consequences on their mental health from such exceptional societal stress as a pandemic constitutes in order to form a hypothesis for how to preserve older people’s mental health during similar future events.

### Supplementary Information

**Supplementary Material 1.**

**Supplementary Material 2.**

## Data Availability

Data are available on request to Linköping University Electronic Press https://ep.liu.se.

## Abbreviations

GDS 20: Geriatric Depression Scale 20 items
HADS: Hospital Anxiety and Depression Scale
HADS-A: Hospital Anxiety and Depression Scale – Anxiety
HADS-D: Hospital Anxiety and Depression Scale – Depression
HOLD: Healthy OLD people
PHC: Primary Health Care center
PSS 10: Perceived Stress Scale 10 items
