# Supplementary material for: Factors affecting self-perceived mental health in the general older population during the COVID-19 pandemic: a cross-sectional study
Source: BMC Public Health. 2024 Mar 1;24:660. doi: 10.1186/s12889-024-18199-1 (PMC10905926; doi:10.1186/s12889-024-18199-1)
Supplement: Supplementary file 1 — Supplementary Material 1. [file 12889_2024_18199_MOESM1_ESM.docx]

***Table S1. Mental health issues and self-reported effect on mental health by the Covid -19 pandemic.***

| **Variables** | ***Yes, affected*** | | ***Do not know if affected*** | |  |
| --- | --- | --- | --- | --- | --- |
|  | **N** | **Mean rank** | **N** | **Mean rank** | **P-value** |
| **GDS-20** | 24 | 29.2 | 30 | 26.2 | 0.48 |
| **HADS depression** | 24 | 26.7 | 30 | 28.1 | 0.74 |
| **HADS anxiety** | 24 | 28.7 | 30 | 26.5 | 0.61 |
| **PSS 10** | 24 | 26.1 | 30 | 28.6 | 0.56 |

*Comparison between self-reported negative effect on mental health by Covid -19 pandemic and rating scales detecting mental health issues in the study population, (divided into: yes, affected; do not know if affected) and Geriatric Depression Scale 20 items (GDS-20), Hospital Anxiety and Depression scale (divided into HADS depression and HADS anxiety), and Perceived Stress Scale 10-items (PSS-10). Total scores were used for all screening scales. Mann Whitney-U test was used for analyses.*
