# Supplementary material for: Factors affecting self-perceived mental health in the general older population during the COVID-19 pandemic: a cross-sectional study
Source: BMC Public Health. 2024 Mar 1;24:660. doi: 10.1186/s12889-024-18199-1 (PMC10905926; doi:10.1186/s12889-024-18199-1)
Supplement: Supplementary file 2 — Supplementary Material 2. [file 12889_2024_18199_MOESM2_ESM.docx]

***Table S2. Correlation seen among the mentally affected group compared to the non-affected group regarding relevant factors during COVID-19. Yes, affected group (n= 24) and No, not affected group (n=204)***

| **Variables** | **Rho-value** | **p-value** | **Number (N=260)** |
| --- | --- | --- | --- |
| **Sex** | 0,171 | 0.01 | 228 |
| **Civil status** | 0,090 | 0.18 | 227 |
| **Education** | 0,067 | 0.32 | 224 |
| **Economic status** | 0,046 | 0.49 | 226 |
| **Family situation** | 0,380 | <0,001 | 226 |
| **Changes in loneliness during COVID -19** | 0,199 | 0.002 | 228 |
| **Changes in social life during COVID-19** | 0,320 | <0,001 | 227 |
| **Changes in physical activity during COVID-19** | 0,353 | <0,001 | 228 |
| **GDS-categories** | 0,169 | 0.01 | 227 |
| **HAD-depression categories** | 0,077 | 0.25 | 227 |
| **HAD-anxiety categories** | 0,299 | <0,001 | 227 |
| **PSS-10 categories** | 0,140 | 0.04 | 227 |

*Spearman’s correlation analyses were used.*
